# Antimicrobial effects of clindamycin-loaded platelet-rich fibrin (PRF)

**DOI:** 10.1007/s00784-024-05532-6

**Published:** 2024-02-13

**Authors:** Anton Straub, Maximilian Stapf, Chiara Utz, Andreas Vollmer, Julia Flesch, Alexander Kübler, Oliver Scherf-Clavel, Thiên-Trí Lâm, Stefan Hartmann

**Affiliations:** 1 Department of Oral and Maxillofacial Plastic Surgery, University Hospital Würzburg, Pleicherwall 2, 97070 Würzburg, Germany; 2 Institute for Pharmacy and Food Chemistry, University of Würzburg, Am Hubland, 97074 Würzburg, Germany; 3 Department of Pharmacy, University of München, Butenandtstraße 5, 81377 Munich, Germany; 4 Institute for Hygiene and Microbiology, University of Würzburg, Josef-Schneider-Straße 2/E1, 97080 Würzburg, Germany

**Keywords:** Agar diffusion test, Antibiotic, Infections, Jawbone, Local application, PRF

## Abstract

**Objectives:**

Recent research has demonstrated that platelet-rich fibrin (PRF) is an appropriate carrier for ampicillin/sulbactam. The aim of the study was to investigate whether PRF is also a suitable bio-carrier for clindamycin (CLI).

**Methods:**

PRF membranes were produced from 36 patients receiving intravenous therapy with CLI (e.g. due to the diagnosis of an osteonecrosis of the jaw or infections). Concentrations of CLI in PRF membranes were measured with liquid chromatography-tandem mass spectrometry, and the antimicrobial effects were investigated in vitro in agar diffusion tests with fresh PRF and PRF stored for 24 h. Storage was performed in an incubator at 36 °C to simulate the in-vivo situation.

**Results:**

The mean concentration of CLI in plasma was 1.0 ± 0.3 μg/100 mg plasma; in resulting PRF membranes 0.7 ± 0.4 μg/100 mg PRF. Agar diffusion tests were performed with *Staphylococcus aureus*, *Streptococcus pneumoniae*, *Streptococcus mitis*, *Porphyromonas gingivalis, *and *Fusobacterium nucleatum*. Mean inhibition zones, in mm, for fresh PRF were 17.3, 12.2, 18.8, 17.1, 25.8 and 18.1, 12.7, 19.2, 17.3, and 26.3 for stored PRF, respectively.

**Conclusion:**

The results demonstrate that PRF is a suitable bio-carrier for CLI when administered systemically to patients. The concentration in PRF generated from patients after infusion of 600 mg CLI dose suffices to target clinically relevant bacteria.

**Clinical relevance:**

Using PRF as a carrier for local antibiotic application can prevent infections in oral and maxillofacial surgery. Within the study limitations, the findings could expand the scope of PRF application by adding CLI as a new antibiotic to the spectrum of PRF therapy.

## Introduction

Platelet-rich fibrin (PRF) forms as a separate fraction on centrifugation of a patient’s blood sample. No additives such as anticoagulants are added to the blood, making PRF a purely autologous blood product, which is known to support processes of wound healing through the release of growth factors. Among others, transforming growth factor beta (TGF-β), platelet-derived growth factor (PDGF), vascular endothelial growth factor (VEGF) and interleukins stimulate cell migration, differentiation and modulate the immune response [1–3]. The fibrin matrix of PRF gives the product a firm structure and allows the release of growth factors over multiple days [4–6].

In addition, the mechanical properties of PRF have other positive effects. For example, it acts as a barrier in oral surgery after alveolar ridge preservation or after the surgical treatment of osteonecrosis of the jaw (ONJ) to improve mucosal closure [7]. It is worth noting that the barrier function of a PRF membrane is not comparable to resorbable collagen membranes. While these collagen membranes remain stable in situ for several weeks, the barrier function of PRF is shorter. Therefore, it is well suited, for example for covering sharp bone edges in cases of ONJ or after augmentation, in addition to a collagen membrane or a titanium mesh but not instead of them [8]. In patients with ONJ, the application of PRF could be beneficial. ONJ is characterized by exposed bone to the oral cavity and is normally treated by surgical necrosectomy and mucosal closure. Wound healing often faces impairments like dehiscences and re-exposure of the bone to the oral cavity. Thus, patients are receiving prolonged, calculated antibiotic therapy, which covers bacteria like Streptococci that are regularly found in necrotic bone samples of patients with ONJ [9, 10]. It can be assumed that the biological and mechanical properties of PRF could support the impaired wound healing in these patients.

Recent research revealed that PRF could be used as a bio-carrier for several drugs and especially for antibiotics [11]. The drugs were added to the blood or the PRF product before or after the centrifugation process [12, 13]. The advantages and disadvantages of these procedures have been discussed elsewhere [14]. Furthermore, PRF demonstrates antimicrobial properties without any further workup or addition of any antibiotic drug, owing to the release of cytokines and enzymes [15–18]. These antimicrobial effects could prevent wound healing disorders especially in patients with ONJ, where antibiotic therapy is a crucial part of the therapy.

In a previous study, it was established that systemically applied drugs are contained within PRF when sufficient plasma concentrations were reached. It is thus possible to load PRF with ampicillin/sulbactam when administered intravenously before blood sampling for PRF production. The antibiotics were released, comparable to the growth factors, over several days in efficacious concentrations. Single-shot application of ampicillin/sulbactam was sufficient to reach concentrations in PRF high enough to be effective. In cases of systemic antibiotic therapy, the beneficial effect of PRF resulting from the release of growth factors can be combined with local antibiotic administration to prevent wound healing disorders [14]. This could be beneficial, for example in the case of alveolar ridge augmentation or in patients with ONJ, but there is a lack of studies investigating the antimicrobial effects of antibiotic-loaded PRF. PRF may be implemented in solid or liquid form, though it remains unclear as to whether solid or liquid PRF is beneficial when used as a bio-carrier for antibiotics. There is currently little evidence indicating any advantage of liquid PRF over solid PRF [19].

Given the incidence of allergy to penicillin, alternative antimicrobial substances such as clindamycin (CLI) are often used [20]. Thus, the aim of the study was to investigate whether PRF membranes are a suitable bio-carrier for CLI when administered intravenously prior to PRF production.

## Materials and methods

### Study design

This in-vitro investigation was conducted at the Department of Oral and Maxillofacial Plastic Surgery of the University Hospital in Würzburg between April 2022 and January 2023. The objective of the study was to investigate whether PRF can be loaded with CLI when it is administered intravenously to the patient. Therefore, the CLI concentration in plasma and PRF was examined by liquid chromatography-tandem mass spectrometry (Bioanalysis of plasma and PRF). Furthermore, the study aimed to investigate whether the CLI concentration in PRF is sufficient to inhibit the growth of bacteria, which are relevant for infections in oral and maxillofacial surgery. This was investigated in agar diffusion tests (Agar diffusion test with CLI-loaded PRF membranes).

**Fig. 1:**
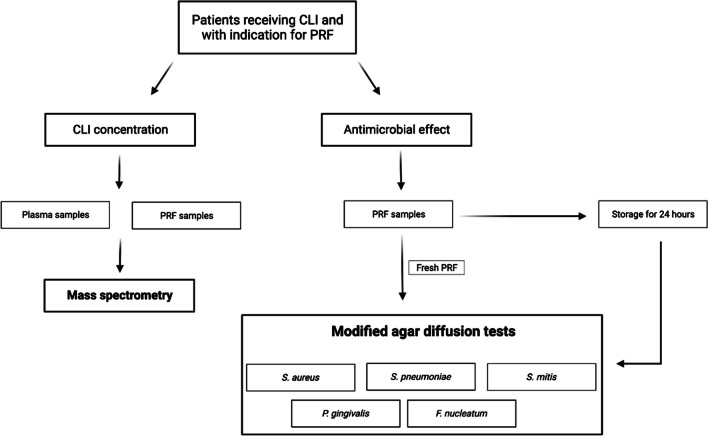
Flowchart: Patients undergoing intravenous therapy with CLI and having an indication for PRF application were included. Plasma and PRF samples were collected. Plasma and a portion of the PRF samples were further processed to investigate the CLI concentration. Another portion of the PRF membranes was further processed to complete modified agar diffusion tests, which were performed with five different bacteria and measuring the inhibition zones after 24 h. The rest of the PRF membranes were stored in an incubator at 36 °C for 24 h and then further processed for an additional set of agar diffusion tests

To be included, patients needed to be aged 18 years or more, on intravenous CLI administration (see below) either as therapy or prophylaxis, as well as have PRF application indicated. CLI was administered due to a disease (ONJ, osteomyelitis, abscesses) that required antibiotic treatment. Patients were excluded from the trial if they were allergic to CLI or when they failed or were unable to comply with study protocols after being included (e.g. neither plasma nor PRF could be obtained). The workflow is depicted in Fig. 1.

Before blood sampling for plasma and PRF, study participants received at least three infusions of CLI (Sobelin® Solubile, Pfizer Pharma GmbH, Berlin, Germany) at a dose of 600 mg every eight hours with an additional infusion administered immediately prior to blood sampling (perioperative prophylaxis). PRF membranes were subsequently produced (Sample collection and preparation) either for mass spectrometry (determination of the CLI concentration) or for agar diffusion tests (antimicrobial effect).

The protocols implemented in this study were approved by the independent review board of the University of Würzburg (IRB approval numbers “51/20-me” and “143/20-me”). The study was conducted in accordance with the Declaration of Helsinki. All patients provided written informed consent.

### Descriptive data

Age, sex and the diagnosis for CLI administration of each participant were taken from the patient’s record. Renal function (glomerular filtration rate) was analyzed prior to surgery. Furthermore, the time between the end of the CLI infusion and the blood sampling for PRF was documented.

### Sample collection and preparation

Venous blood samples were drawn intraoperatively by venipuncture using 1.6 mL EDTA tubes (S-Monovette, Sarstedt, Sarstedt-Straße 1, 51588 Nümbrecht, Germany) 5–20 min after the end of infusion. Blood samples were then centrifuged at 4900 rpm for 10 min at 4 °C (Hettich Universal 320 R, Andreas Hettich GmbH & Co.KG, Tuttingen, Germany). The obtained plasma was separated from the cellular components and divided into four aliquots of 100 μL each. Aliquots were stored at − 80 °C until the day of analysis.

Blood sampling for PRF was performed together with blood plasma collection using at least four and up to six sterile vacuum PRF tubes (Dr. Choukron Glass A-PRF Tubes, 10 mL, Process for PRF, Nice, France) and centrifuged with a Duo Quattro centrifuge (PRF, Nice, France) at 2300 rpm for 12 min and RCFmax = 652 g. Samples were stored at − 80 °C for further processing in the Institute for Pharmacy and Food Chemistry of the University of Würzburg or directly processed for agar diffusion tests in the Institute for Hygiene and Microbiology of the University of Würzburg. Due to a limited amount of PRF membranes, it was not possible to proceed membranes of each patient for mass spectrometry (Bioanalysis of plasma and PRF) and agar diffusion tests (Agar diffusion test with CLI-loaded PRF membranes). Therefore, membranes acquired from different patients were used for the two methods.

### Bioanalysis of plasma and PRF

Plasma and PRF samples were processed using protein precipitation. Acetonitrile was precipitating agent for plasma, whereas 80% methanol was used as the agent for PRF. The final prepared plasma and PRF samples were then analyzed by liquid chromatography-tandem mass spectrometry (LC–MS/MS) at the Institute for Pharmacy and Food Chemistry of the University of Würzburg, using a validated bioanalytical method [21]. CLI was monitored through electrospray ionization in multiple-reaction-monitoring (MRM) mode. Here, the positive-ion mode was applied. The method used isotopically labelled clindamycin (CLI-D3) as internal standard. MRM transitions used for quantification were m/z 425.1 → 126.0 for CLI and 428.2 → 129.1 for CLI-D3. The method was successfully validated in terms of sensitivity, linearity, selectivity, carryover, within-run and between-run accuracy and precision, matrix effect, extraction recovery and stability in both matrices.

### Agar diffusion test with CLI-loaded PRF membranes

To investigate the antimicrobial effects of CLI-loaded PRF, modified agar diffusion tests were performed on the EUCAST disc agar diffusion methodology with *Staphylococcus aureus* ATCC 29213; *Streptococcus pneumoniae* ATCC 49619; *Streptococcus mitis* DSM 12043; *Porphyromonas gingivalis* ATCC 33277; and *Fusobacterium nucleatum* InHM 39, a clinical isolate [22]. As there are no testing guidelines from EUCAST for *P. gingivalis*, *S. mitis,* and *F. nucleatum*, preliminary tests were performed to establish a suitable test protocol based on procedures according to EUCAST. Bacterial suspensions for *S. aureus*, *S. pneumoniae,* and *S. mitis* were adjusted to a McFarland turbidity standard of 0.5 in 0.85% saline using a DensiCHEK Plus (bioMérieux, Nürtingen, Germany). The bacterial suspensions for *P. gingivalis* and *F. nucleatum* were adjusted to a McFarland turbidity standard of 1.0 using the same procedure. *Streptococcus aureus* inocula were plated on unsupplemented Mueller–Hinton E agar (MH-E, bioMérieux, Nürtingen, Germany), *S. pneumoniae* and *S. mitis* were plated on Mueller–Hinton agar containing 5% defibrinated horse blood and 20 mg/L β-NAD (MH-F, BD, Heidelberg, Germany). *Porphyromonas gingivalis* and *F. nucleatum* were plated on Brucella Blood Agar with Hemin and vitamin K1 (BD Brucella Blood Agar with Hemin and vitamin K1, Heidelberg, Germany). The McFarland-adjusted bacterial suspension was spread evenly over the entire surface of the agar plate using a cotton swab. A 6-mm PRF disc (protocol see above) was placed on each inoculated plate and incubated for 24 h. Ten experiments for each bacterium were performed. The same experiments were carried out with a 6-mm PRF disc stored at 36 °C for 24 h before being placed on the inoculated plates for a further 24 h (stored PRF).

A number of technical controls were used. Firstly, a disc agar diffusion test was completed using an antimicrobial susceptibility test disc (ThermoScientific Oxoid, Langenselbold, Germany) loaded with 2 μg CLI. Secondly, a gradient agar diffusion test was performed in parallel using a test strip (Liofilchem, Roseto degli Abruzzi, Italy) loaded with a gradient ranging from 0.016 to 256 mg/L of CLI. Agar plates were incubated at 36 °C for 24 h in ambient air (*S. aureus*) or at 35 °C in 5% CO_2_ atmosphere (*S. pneumoniae*, *S. mitis*).

Agar plates containing *F. nucleatum* were stored in an anaerobic box with a GENbox anaerobic bag (bioMérieux, Nürtingen, Germany) and dry BD-BBL anaerobic indicator strips (BD, Heidelberg, Germany) for 48 h at 36 °C until read-out was completed. Agar plates containing *P. gingivalis* were stored in the same manner, but were evaluated after 7 days. Upon incubation, the diameters of the inhibition zones (IZ) were measured in millimetres and photographs were taken for documentation purposes.

### Data processing and statistics

Descriptive statistical analyses were performed with GraphPad Prism (Version 10, San Diego, USA). Differences between the means were examined using paired, two-tailed and non-parametric Wilcoxon test if the Shapiro–Wilk normality test was significant (*p* < 0.05). If the Shapiro–Wilk test was insignificant, differences between the means were examined using paired and two-tailed Student’s *t*-test. Statistical significance was considered if *p*-values were less than 0.05 (*p* < 0.05). Correlations were examined with non-parametric Spearman’s rho test. All statistical analyses were performed with GraphPad Prism (Version 10, San Diego, USA). The resulting data is expressed as means and standard deviations.

## Results

We included 36 patients in this prospective trial. The mean age was 70 years (SD ± 9.1 years) with 19 men and 17 women. Plasma and PRF samples were obtained from 26 of the 36 patients and further processed for CLI concentration determination. PRF samples alone were obtained from a further ten patients and processed for agar diffusion tests. The mean time interval between the end of the final CLI infusion and blood collection for plasma and PRF samples was 15.5 min. Further descriptive statistics are portrayed in Table 1.

**Table 1:** Patient characteristics

	*N* = 36*
Mean age (in years) Min and max age	70 (SD ± 9.1) 49–85
Sex (m/f)	19/17
Diagnosis	
MRONJ	26
ORN	2
OM	2
Others	6
Time after infusion**	15.5 (SD ± 6.0) min
Renal function***	78.1 ± 29.9 mL/min*

*N*, number of participants; *m*, male; *f*, female; *ORN*, osteoradionecrosis; *MRONJ*, medication-related osteonecrosis of the jaw; *OM*, osteomyelitis

^*^All in all, 36 patients were included in this study. PRF samples of 26 patients were processed for investigation of CLI concentration in plasma und PRF, and samples of a further 10 patients were processed for agar diffusion tests

^**^Mean time gap between the end of CLI infusion and blood sampling for PRF

^***^Glomerular filtration rate (MDRD) in mL/min

**Fig. 2:**
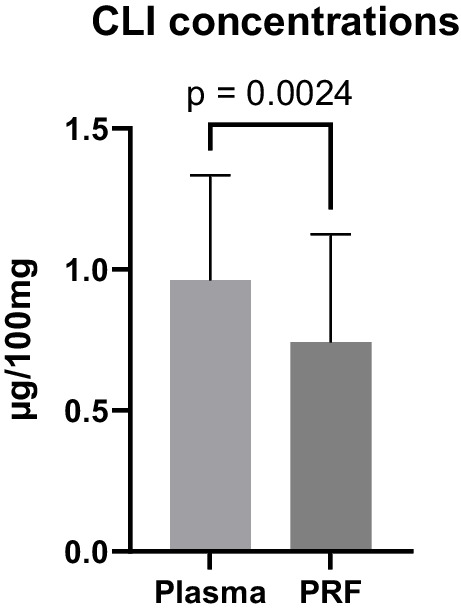
CLI concentrations in plasma per 100 mg and in the solid PRF membranes per 100 mg. Concentrations were adjusted to /100 mg because an average PRF membrane had a mass of 100 mg. Error bars represent the standard deviation

Determination of the antibiotic concentration in plasma revealed a mean of 10.0 μg/mL CLI, which was converted to μg/100 mg plasma for a better comparison with the PRF membranes assuming a plasma density of 1 g/mL. The mean concentration of CLI was 0.7 μg/100 mg in PRF, which was significantly lower than in plasma (Wilcoxon rank test *p* < 0.05, see Table 2 and Fig. 2).

**Table 2:** Concentrations of CLI in plasma and solid PRF membranes

	Plasma in μg/mL	Plasma μg/100 mg	PRF μg/100 mg
Concentration	10.0	1.0	0.7
SD	± 3.3	± 0.3	± 0.4
95% CI	8.6–11.3	0.8–1.1	0.6–0.9
Minimum	3.4	0.3	0.4
Maximum	19.1	1.9	2.3

*SD*, standard deviation; *95% CI*, 95% confidence interval

**Fig. 3:**
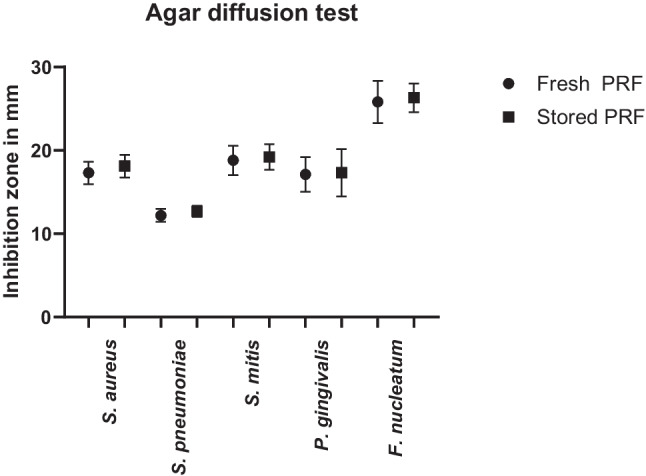
Mean diameter of the IZs for fresh PRF and for stored PRF. Bars represent the standard deviation

The mean IZ in agar diffusion tests using fresh PRF in plates inoculated with *S. aureus*, *S. pneumoniae*, *S. mitis*, *P. gingivalis,* and *F. nucleatum* ranged from 12.2 to 25.8 mm (see Table 3 for details). For stored PRF IZs of 18.1, 12.7, 19.2, 17.3, and 26.3 mm for *S. aureus*, *S. pneumoniae*, *S. mitis*, *P. gingivalis,* and *F. nucleatum*, respectively, were measured. Standard deviations and ranges of the values are summarized in Table 3 and Fig. 3.

**Table 3:** Size of the inhibition zones of a 6 mm PRF disc in agar diffusion tests

*N* = 10		*S. aureus*	*S. pneumoniae*	*S. mitis*	*P. gingivalis*	*F. nucleatum*
Fresh PRF	IZ	17.3	12.2	18.8	17.1	25.8
	SD	± 1.3	± 0.8	± 1.8	± 2.1	± 2.5
	Range	15–20	11–13	16–22	14–21	23–32
Stored PRF	IZ	18.1	12.7	19.2	17.3	26.3
	SD	± 1.4	± 0.7	± 1.5	± 2.8	± 1.7
	Range	16–21	12–14	17–22	13–21	23–29
*p*		0.2	0.1	0.6	0.8	0.6

*IZ*, inhibition zones in mm; *SD*, standard deviation; *p*, *p*-value for comparison between fresh and 24-h storage condition (*t*-test, paired and two-tailed, significance *p* < 0.05)

IZ and ranges for stored PRF were slightly higher than for fresh PRF, but differences were not statistically significant (see Table 3 for the corresponding *p*-values).

No statistical correlation between time after infusion and the size of the IZ (Spearman *r* = 0.4, *p* = 0.28) was found. Furthermore, there was no statistical correlation between renal function and the size of the IZ (Spearman *r* =  − 0.09, *p* = 0.81).

## Discussion

To our knowledge, this is the first study investigating the antibiotic concentration of CLI within PRF and its antimicrobial effects. Previous studies revealed that PRF can function as a bio-carrier for drugs in general and antibiotics in particular. Drugs may be administered to the patient before the blood sample is taken, to the patient’s blood before centrifugation, and directly to the PRF product after the centrifugation process [12, 14, 23, 24]. Both solid and liquid PRF can be used as a drug delivery system [11]. If antibiotic treatment is indicated, for example as perioperative prophylaxis, no further treatment or workup of the blood or PRF product is necessary to load it with the administered antibiotic [14]. In this way, a local drug carrier can be generated by choosing the right time for blood collection. The distribution time defines the time it takes for drug concentrations in plasma to peak and remain equal throughout the whole body. Data indicate that concentrations within PRF are highest when the sample is drawn directly after the distribution time has passed (and no later), which has been determined to be only a few minutes in duration [25]. While perioperative prophylaxis lasts only a few hours, an antimicrobial effect with PRF can probably be achieved for 48 h [26]. The indication for antibiotic therapy determines which drug is administered and accumulates in the PRF product accordingly. The addition of a drug to the drawn blood or the generated PRF product is more flexible and does not depend on the treatment indication, as it is not given to the patient, and any desired preparation can be added. On the other hand, this presents a risk in cases of substance intolerance or allergies. Nevertheless, perioperative prophylaxis in the form of empirical antibiotic therapy should cover the bacteria expected at the operation site [27].

In this study, patients received CLI if they were allergic to ampicillin/sulbactam. A dose of 600 mg CLI was administered three times daily and the last dose directly before the blood sample for PRF and plasma samples was drawn. Compared to a previous study of the authors, which revealed equally high concentrations of ampicillin/sulbactam in PRF and plasma, results of this study revealed a significantly lower concentration within PRF compared to patients’ plasma (*p* < 0.05) [14]. CLI is known to have higher plasma protein binding (approx. 60–94%) compared to ampicillin/sulbactam (approx. 20%), which could explain this observation [28, 29]. As concentrations of CLI were sufficient to reach a bacteria-inhibiting effect in agar diffusion tests, we think that this statistical significance is not of clinical relevance.

The antimicrobial effect of PRF preloaded with antibiotics, in addition to the release of growth factors, could expand the scope of PRF application. In periodontal therapy, for example the benefit of applying growth factors through PRF has already been reported and antibiosis is known to be effective in periodontal patients [30–32].

Furthermore, it was demonstrated in a previous study that the antibiotic concentration in bone tissue is significantly lower than in plasma in patients with ONJ. Concentrations for ampicillin/sulbactam were lower by a factor of 20. For CLI, unpublished work by Straub et al. demonstrates that bone concentrations were lower by a factor of 5 compared to the corresponding plasma concentrations [21, 26, 33]. Thus, in vital bone samples of patients with MRONJ, a mean CLI concentration of 2.3 μg/g (SD ± 1.4 μg/g) was measured. This corresponds to an adjusted CLI concentration of 0.23 μg/100 mg and is one-third of the CLI concentration measured in PRF [26]. A study of 31 patients by Mueller in 1999 revealed CLI bone tissue concentrations ranging from not detectable to 3.4 mg/L (0.34 μg/100 mg). This study provided neither information regarding the mean CLI bone tissue concentration nor the disease nor the indication for antibiotic treatment. The maximum CLI concentration in the study by Mueller was still lower than the mean measured antibiotic concentration in PRF in this study, which supports the local application of PRF in patients undergoing jaw surgery [34]. Comparing concentrations in the present study with previous studies is difficult, because antibiotic dosing schemes and/or routes of application as well as the chosen methods of quantification differ between studies.

Considering streptococci, staphylococci, and other bacteria normally present in the oral cavity, the minimum inhibitory concentration (MIC) is below 0.5 mg/L (corresponding to 0.05 μg/100 mL) and therefore lower than the CLI concentration in PRF [10, 35, 36]. Concentrations within PRF are also sufficient to combat anaerobic bacteria (MIC approx. 2 mg/L corresponding to an adjusted MIC of 2 μg/100 mL), which could indicate that application of PRF preloaded with antibiotics in patients after jaw surgery is beneficial [35, 37]. Additionally, antibiotic-loaded PRF can probably achieve a longer lasting antimicrobial effect than a single CLI application. According to the data published in the study by Mueller et al., the plasma concentration of CLI falls from 12.73 to 1.41 mg/L within 8 h after a single-dose administration and is probably insufficient to combat most bacteria a few hours later [34], while the stored PRF in the present study still exerts an antimicrobial effect after 48 h.

Comparing the mean IZ in agar diffusion testing during this study with that of the previous study by the authors, in which PRF was loaded with ampicillin/sulbactam, the IZ diameter was smaller for *S. aureus* and *S. pneumoniae* (CLI vs ampicillin/sulbactam, 17.3 mm vs 19.6 mm and 12.2 mm vs 28.4 mm). Other bacterial strains (*H. influenzae* and *E. coli*) were not tested in this study, because CLI is known to be ineffective against these bacteria, and therefore could not be compared [14]. CLI is thus only employed as alternative therapy in cases of allergy to penicillin.

Further investigations will be necessary in order to receive more information regarding the release of CLI out of PRF. A limitation of this study is the storage of PRF in an incubator to simulate the in-vivo situation. Further research should focus on the release kinetics and establish realistic in-vitro models to gain further insight into the duration of the antimicrobial effect of PRF. Furthermore, most bacteria in the oral cavity grow in biofilms; single bacteria strains do not reflect the clinical situation correctly. The MICs of bacteria in biofilms are known to be higher than of single strains, which could reduce the clinical efficacy of both systemic and local antibiotic therapy [38]. However, the study demonstrates that antibiotic-loaded PRF reaches concentrations in vitro comparable to the plasma concentration. As mentioned previously, CLI is not effective against Gram-negative bacteria such as *E. coli* and *H. influenzae*, in contrast to ampicillin/sulbactam. This should be reflected when the decision for empirical antibiotic therapy or prophylaxis is made. Furthermore, we included patients suffering from different diseases like ONJ, osteomyelitis, and abscesses. This could influence the properties of PRF as a bio-carrier for antibiotics. However, we think that it is more likely that these diseases influence the content and release of growth factors out of PRF rather than the mechanical and antibiotic properties.

## Conclusions

The results of this study revealed new information about the antimicrobial effect of PRF in an in-vitro setting, which may expand the indication for the application of PRF: In summary, PRF may be used as a matrix for drugs and loaded with CLI when it is administered intravenously to the patient before blood sampling for PRF is performed. Concentrations of CLI in PRF were probably higher than in the bone tissue and sufficient to inhibit the growth of most bacterial species present in the oral cavity. Furthermore, the antimicrobial effect of PRF preloaded with CLI appears to last longer than that of a single dose of CLI administered as bolus. From a clinician’s perspective our results indicate, that a CLI-loaded PRF might support wound healing in compromised patients by local antibiotic delivery.

## Data Availability

No datasets were generated or analysed during the current study.
